# The Need for Testing—The Exercise Challenge Test to Disentangle Causes of Childhood Exertional Dyspnea

**DOI:** 10.3389/fped.2021.773794

**Published:** 2022-01-06

**Authors:** Vera S. Hengeveld, Mattiènne R. van der Kamp, Boony J. Thio, John D. Brannan

**Affiliations:** ^1^Department of Pediatrics, Medisch Spectrum Twente, Enschede, Netherlands; ^2^Department of Biomedical Signals and Systems, University of Twente, Enschede, Netherlands; ^3^Ludwig Engel Centre for Respiratory Research, Westmead Institute for Medical Research, Westmead, NSW, Australia; ^4^Department of Respiratory and Sleep Medicine, John Hunter Hospital, New Lambton Heights, NSW, Australia

**Keywords:** asthma, exercise induced bronchoconstriction, dyspnea, child, physiological limit, dysfunctional breathing, spirometry and other lung function tests, exercise test

## Abstract

Exertional dyspnea is a common symptom in childhood which can induce avoidance of physical activity, aggravating the original symptom. Common causes of exertional dyspnea are exercise induced bronchoconstriction (EIB), dysfunctional breathing, physical deconditioning and the sensation of dyspnea when reaching the physiological limit. These causes frequently coexist, trigger one another and have overlapping symptoms, which can impede diagnoses and treatment. In the majority of children with exertional dyspnea, EIB is not the cause of symptoms, and in asthmatic children it is often not the only cause. An exercise challenge test (ECT) is a highly specific tool to diagnose EIB and asthma in children. Sensitivity can be increased by simulating real-life environmental circumstances where symptoms occur, such as environmental factors and exercise modality. An ECT reflects daily life symptoms and impairment, and can in an enjoyable way disentangle common causes of exertional dyspnea.

## Introduction

Exertional dyspnea is a common presenting symptom within the pediatric population; up to 14% of the adolescent population experience exercise-induced dyspnea yearly (1). Exertional dyspnea can be detrimental for children, impairing participation in play and sports. According to WHO-guidelines, all children and adolescents should exercise daily for 60 min or more with moderate-to-vigorous intensity (2). Only around half of the Dutch pediatric population tend to adhere to these guidelines, whereas Hardy et al. (3) showed an even lower adherence among Australian children (3, 4). To attain sufficient physical activity it is critical that a child can enjoy play and sports and does not experience restraining symptoms such as dyspnea. Regular physical activity is paramount for the development of children since it not only promotes cardiorespiratory fitness and muscle strength, but also offers opportunities for self-expression, building confidence and social interaction and integration (2).

Refraining from sports and play with moderate-to-vigorous intensity will lead to physical deconditioning. This can lead to premature drop out of exercise with peers and worsening of cardiopulmonary fitness and exertional dyspnea. In asthmatic children there is also increasing evidence that physical deconditioning can lead to aggravation of asthma severity and further increase of exertional dyspnea in asthmatic children (5, 6). In addition, decreased physical activity contributes to changes in body composition since it can lead to reduced muscle mass and/or an increased deposition of adipose tissue. This is not only a burden for the cardiopulmonary system but also increases pro-inflammatory tissue, associated with increased asthma severity (7–11). To break through these self-perpetuating circles, it is important to identify the correct cause(s) of exertional dyspnea and reverse them, to enable children to be physically active without symptoms.

### Exertional Dyspnea—Coexisting Diagnoses

Dyspnea is a subjective phenomenon, influenced by interacting physiological and psychological factors, along with social and environmental input (12). Exertional dyspnea has an extensive differential diagnosis (Table 1). Exertional dyspnea in children, especially when having a history of asthma, is often primarily assumed to be caused by exercise induced bronchoconstriction (EIB), as asthma is a common and known entity in childhood. Other prevalent, but less known causes of exertional dyspnea are dysfunctional breathing, Exercise Induced Larygneal Obstruction (EILO) and dyspnea when reaching the physiological limit (13–18). Abu-Hasan et al. showed that the perception of dyspnea when reaching the physiological limit seems the most prevalent cause of childhood exertional dyspnea, by reviewing all 142 ECT's performed because of exertional dyspnea in their pediatric clinic. Dyspnea at reaching their physiological limit was the main cause in 52% of children, sometimes aggravated by physical deconditioning, indicating that in the majority of children there is mainly an issue with the perception of dyspnea, rather than a sign of underlying pathology (19).

**Table 1:** Common (bold) and rare causes of exertional dyspnea in children, with corresponding red flag signs and symptoms.

	**Diagnosis**	**Red flags**
Respiratory	Low airway obstruction **-Asthma/Exercise induced bronchocontriction** -Vascular ring -Stenosis or airway malacia -Tumor	
	Upper airway obstruction **-Exercise induced laryngeal obstruction** -Corpus alienum	
	**Dysfunctional breathing**	
	Infectious lung diseases	Fever
	Interstitial lung diseases	
	Diaphragma paralysis	
	Pneumothorax	
Cardial	Cardiac shunting -Atrial and/or ventricular septum defect -Significant arteriovenous malformation	Oxygen desaturation
	Arrhythmia	Family history, (pre-)syncope
	Pulmonary hypertension	
	Lung embolism	
	Pericarditis	Fever, chest pain
	Cardiomyopathy	Family history
Neuromuscular	Myasthenia gravis	
Metabolic	Glycogen storage disease (e.g., McArdle)	Sudden muscle cramps
	Mitochondrial enzyme deficiency	
	Thyroid disease	
Other	**Physical deconditioning**	
	**Reaching physiological limit**	
	Ear nose throat pathology	
	Anemia	
	Gastro-oesophagal reflux	

All these common causes of exertional dyspnea regularly coexist, influence one another and have overlapping symptoms which can impede diagnosis and treatment (20). The purpose of this article is to provide an overview of the causes of exertional dyspnea and the diagnostic approach toward the child with exertional dyspnea, which is visualized in the Supplementary Figure 1.

### Exercise Induced Bronchoconstriction

Exercise induced bronchoconstriction (EIB) is a common, highly specific symptom in childhood asthma and is a useful objective marker to indicate poor asthma control (21–24). It is a sign of bronchial hyperresponsiveness caused by airway inflammation and can present with the classic symptoms of asthma, such as dyspnea, wheezing, cough, mucus hyper-secretion, chest tightness and/or nocturnal wakening (25). Reported prevalence of EIB among children ranges between 10 and 20% (26, 27). Important anamnestic cues pointing toward EIB are: a (family) history of asthma and/or allergy, a positive effect of reliever medication pre-exercise or relief post exercise, and negative impact on symptoms of bronchoprovocative conditions such as cold air or allergens. Physical examination can show signs of allergic rhinitis, such as Dennie-Morgan lines or nasal crease. Baseline lung function measured by forced spirometry, is often completely normal in children with EIB (28, 29).

Although an accurate anamnesis is helpful to direct toward the most likely cause(s) of exertional dyspnea, previous research showed that self-reported symptoms (15, 28, 30–33) and questionnaires such as the Asthma Control Questionnaire (34) are unreliable to identify EIB. Even experienced pediatricians could poorly predict occurrence of EIB based on anamnesis, physical examination and lung function alone, as shown by Lammers et al. (35). The diagnosis of EIB should thus never be made based on symptoms and/or reaction on reliever medication alone, but should be accompanied by data on changes in lung function in response to exercise or a surrogate challenge (27, 31).

The goal of therapy for EIB is to prevent symptoms induced by exercise, to enhance overall control of asthma, and to ameliorate symptoms rapidly when they occur (27). Non-pharmacological treatment consists of pre-exercise warm-up, which can help reducing EIB severity. The first step of pharmacological treatment of EIB includes inhaled corticosteroids and beta_2_-agonists as-needed (27, 36).

### Dysfunctional Breathing

Dysfunctional breathing is an umbrella term for abnormal breathing patterns such as a high-thoracal breathing pattern, hyperventilation, periodical deep sighing or thoraco-abdominal asynchrony, that lead to intermittent or chronic symptoms (37). Symptoms of dysfunctional breathing can be pulmonary (short of breath, unable to breathe deeply, faster or deeper breathing) or extra-pulmonary (blurred vision, tingling or stiff fingers, tight feelings round the mouth, palpitations) and can range from mild to severe (37, 38). EIB can trigger dysfunctional breathing and vice versa, and thereby lead to disproportionate symptoms (39). There is no gold standard to diagnose dysfunctional breathing yet, which makes it impossible to accurately determine the prevalence of dysfunctional breathing.

Around 5% of the adolescent population experience an inspiratory airflow limitation during exercise due to inappropriate closure of the larynx: EILO (1, 40). It is a frequent, but underdiagnosed cause of exertional dyspnea that mainly affects adolescent, female athletes (41). EILO can originate from a dysfunctional breathing pattern, but can also have an anatomical substrate. Typical are the inspiratory stridor, occurrence during peak effort and disappearance of complaints shortly after stopping exercise (42).

Although an ECT is primarily designed to diagnose EIB, close observation of breathing patterns during exercise can reveal dysfunctional breathing patterns accompanied by symptoms. When there is a high clinical suspicion of EILO from reported symptoms it is optional to make the subject perform a final peak effort in an attempt to provoke EILO symptoms. EILO can be diagnosed by performing continuous laryngoscopy during exercise (43, 44).

Dysfunctional breathing is commonly treated non-pharmacological by a physiotherapist, speech- and language therapist or psychologist, depending on the dominant features (i.e., abnormal breathing pattern, EILO or combination) (18). Surgical supraglottoplasty has been used to treat a selection of patients with severe supraglottic EILO, however the place for surgery in treatment of EILO has not been settled (45).

### Physiological Limitation and Physical Deconditioning

Normal physiological limitation is the most common cause of exertional dyspnea in children and adolescents (16, 19). Dyspnea at peak workload is a physiological phenomenon, but can nevertheless be misinterpreted as a sign of disease. During physical exercise, an adequate amount of oxygen and nutrients has to be transported to the exercising muscles, and metabolically produced carbon dioxide has to be removed to maintain homeostasis. Increasing exercise intensity results in an increased stroke volume and heart frequency as well as increased tidal volume and breathing frequency (46). Therefore, reaching the physiological limit can be the cause of exertional dyspnea in children with poor as well as excellent cardiovascular condition (16, 19). Physiological limitation, however, is reached much earlier when cardiovascular condition is poor due to increased ventilatory equivalent at smaller workload (47). Significant physical deconditioning can therefore cause excessive exertional dyspnea and early drop-out at a workload that peers can easily handle.

When reaching the physiological limit is the cause of exertional dyspnea, reassurance is often enough. Children with a poor cardiovascular condition can be referred to a physiotherapist for supervised training.

### Exertional Dyspnea—Red Flags

Furthermore, It is important to be aware of “red flags” (such as oxygen desaturation or (pre)-syncope) to identify rare, yet serious, causes of exertional dyspnea, such as cardiovascular, neuromuscular or metabolic disorders that require further investigation (14). Table 1 includes common, but also rare causes of exertional dyspnea and red flags.

## Exercise Challenge Test

**Figure 1:**
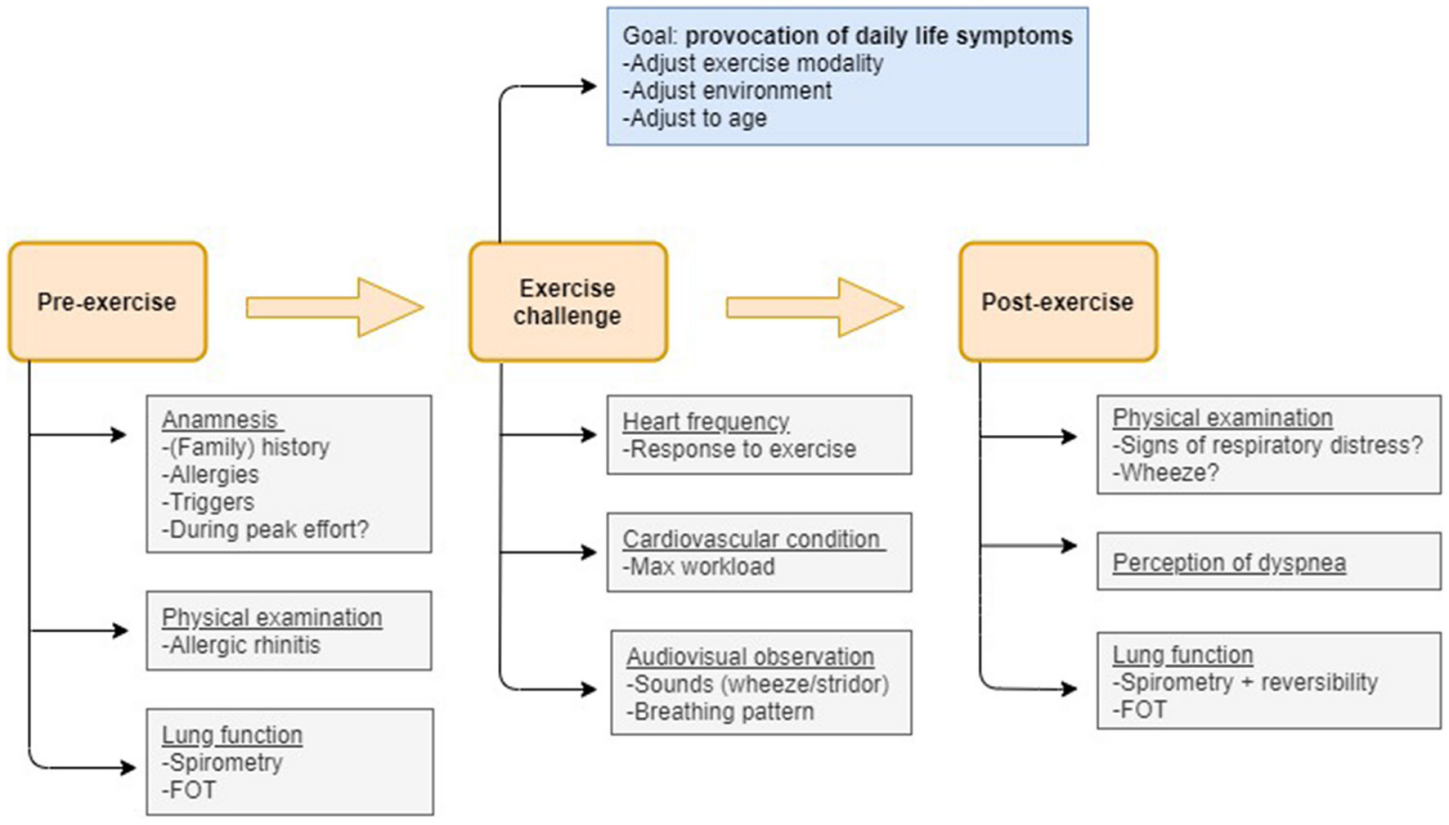
Flow-chart visualizing the measurements and points of attention pre-exercise, during the exercise challenge and post-exercise that allow to disentangle the most prevalent causes of exertional dyspnea in children.

An exercise challenge test (ECT) is a non-obtrusive, real-life test that provides patients, caregivers and medical professionals more insight into the predominant cause(s) of exertional dyspnea. An ECT is an indirect bronchoprovocation test assessing bronchial hyperreactivity to exercise. Subjects perform a submaximal effort during 4–6 min, attempting to provoke recognizable symptoms (48). Lung function is measured at baseline, post-exercise and post-salbutamol. EIB is diagnosed when exercise results in a fall in FEV1 (or FEV0.5) of ≥ 13–15% post-exercise compared to baseline (31). Indirect challenge tests trigger airway narrowing through activation of the endogenous inflammatory pathways involved in asthma. Direct bronchoprovocation challenges (e.g., methacholine) do not activate these pathways but act directly on the airway smooth muscle receptor. They are generally more sensitive but less specific than indirect bronchoprovocation challenges (49, 50). An advantage of an ECT compared to other direct and indirect tests is the possibility to identify other causes of exertional dyspnea besides EIB during one test (19, 51). Figure 1 visualizes the steps and points of attention during the phases of an ECT.

### Exercise Intensity

The intensity of exercise during an ECT can be titrated on heart frequency (85% of maximal) or target ventilation. Monitoring ventilation requires children to wear a face mask or mouthpiece during exercise. This can be frightening, disturb normal breathing patterns and provoke dysfunctional breathing. When necessary, an additional VO2 max test and/or end-tidal pCO2 measurement can be performed to obtain more detailed information about cardiorespiratory condition (15, 52).

It is preferable to simulate the exercise modality that provokes the reported symptoms. Older children (>8 years) can therefore often be tested on a treadmill, especially when performing running sports. Using a slope angle of 10% decreases the running pace required to reach target heart frequency. This asks for less coordination and is therefore safer; if coordination is poor the slope angle can be further increased to reduce running speed. Treadmill speed in relation to heart rate and/or ventilation and age provides an indication of cardiovascular condition. The quick rise in ventilation makes the treadmill a very suitable way of testing, since too long of a warming-up period may decrease the likelihood of identifying mild EIB (48). Alternatives to running are cycle ergometry or a jumping castle.

### Environmental Factors

Environmental factors such as smoke, dust, pollen, swimming pool trichloramines and (changes in) air temperature and humidity can trigger exercise-induced symptoms (53). To elicit the reported symptoms, the real-life environmental conditions where symptoms occur should be simulated. This condition can be individually different but it is often outdoors. Testing in cold and/or dry air enhances the stimulus for bronchoconstriction in asthmatic patients (48). Cold and/or dry air therefore significantly increases sensitivity and repeatability of the ECT for EIB compared to testing at normal room temperature (54). In a climate chamber outdoor conditions can be approximated while the child can exercise without wearing a facial mask or mouthpiece and is therefore preferred. However, when suspicion of EIB is low and symptoms predominantly arise indoors, cold and/or dry air is not compulsory.

### Testing Young Children

Running on a treadmill requires well-coordinated movements and is therefore less suitable for younger children. In this age group (3–6 years) a jumping castle can be used to induce a rapid increase in heart frequency that is sustained over an extended bout length (55). It is a safe, fun and feasible way of testing. Children can learn to perform forced breathing maneuvers when they are as young as 3 years old. The majority of children can perform technically acceptable and repeatable spirometry by the age of six, when instructed by experienced professionals (56–58).

In these young children, FEV0.5 is a more suitable outcome measurement when interpreting spirometry than FEV1, considering the limited duration of their exhalation (57, 59).

The forced oscillation technique (FOT) can be helpful to diagnose EIB, especially when children are not able to perform the forced breathing maneuvers required for spirometry since it requires no active cooperation. FOT uses small-amplitude pressure oscillations superimposed on normal breathing to measure respiratory mechanics (60–62). An exercise-induced transient increase in resistance and decrease in reactance is highly suggestive of EIB. Cut-off values for EIB of >45% increase in resistance and/or >45% decrease in reactance at 5 Hz have been suggested by referencing FOT values to spirometry (58, 63).

### Breakthrough Phenomenon

Peak fall in FEV1 usually occurs 3–15 min after termination of exercise (23, 64). However, the younger the child, the shorter the time to maximal bronchoconstriction and the quicker the recovery from EIB (55, 65). A part of children with EIB even experience bronchoconstriction during exercise, also called “breakthrough”-EIB (BT-EIB). Van Leeuwen showed this phenomenon in 36% of children aged 5–7 years (*n* = 82), with some children showing a significant fall in FEV1 after only 2 min of exercise. BT-EIB is characterized by an earlier and often also deeper fall in FEV1, and can therefore be considered a more severe form of EIB which is detrimental for athletic performance (66). The rapid recovery of EIB in children, especially in younger children, underlines the importance to start spirometry measurements shortly after exercise (preferably 1 min after termination of exercise), because otherwise bronchoconstriction can be missed!

## Conclusion

Exertional dyspnea is a common symptom in childhood which can induce avoidance of physical activity, aggravating the original symptom. Common causes of exertional dyspnea, such as EIB, dysfunctional breathing, physical deconditioning and reaching the physiological limit frequently coexist, trigger one another and have overlapping symptoms, which can impede diagnoses and treatment. Although often primarily assumed, exertional dyspnea is not caused by EIB in the majority of children (19, 67). Self-reported symptoms and questionnaires are unreliable to diagnose EIB as children exhibit a large variation in the perception of dyspnea symptoms (34, 35, 68). The diagnosis of EIB should thus never be made based on symptoms alone, but be accompanied by data on changes in lung function in response to exercise or a surrogate challenge (27, 31).

An ECT is a real-life test that reflects daily life symptoms and impairment, and can be used to disentangle the most prevalent causes of exertional dyspnea. With the proper adjustments, children can perform an ECT when they are as young as 3–6 years old. Sensitivity of testing can be increased by simulating real-life circumstances, such as exercise modality, exercise intensity and environmental factors, in which dyspnea normally occurs Especially when symptoms predominantly arise at cooler temperatures than ambient air, testing in cold or dry air is needed to exclude EIB as a contributor to exertional dyspnea.

## Take-home messages

1) Avoidance of physical activity due to exertional dyspnea can aggravate symptoms over time.
2) In the majority of children with exertional dyspnea EIB is not the cause.
3) An ECT is a real-life test that can disentangle the most prevalent causes of exertional dyspnea in children.

## Data Availability Statement

The original contributions presented in the study are included in the article/Supplementary Material, further inquiries can be directed to the corresponding author.

## Author Contributions

BT, MK, and VH conceived the original idea. VH wrote the draft manuscript with input from MK, BT, and JB. BT and JB provided critical feedback to the draft manuscript several times. VH and MK processed this feedback together to create the final manuscript. All authors contributed to the article and approved the submitted version.

## Author Disclaimer

All views expressed within this manuscript are original and are based upon findings of the authors. None of the views expressed are of any of the above listed institutions.

## Conflict of Interest

The authors declare that the research was conducted in the absence of any commercial or financial relationships that could be construed as a potential conflict of interest.

## Publisher's Note

All claims expressed in this article are solely those of the authors and do not necessarily represent those of their affiliated organizations, or those of the publisher, the editors and the reviewers. Any product that may be evaluated in this article, or claim that may be made by its manufacturer, is not guaranteed or endorsed by the publisher.

## Supplementary Material

Supplementary Figure 1 Diagnostic pathway for childhood exertional dyspnea.
