# Supplementary figures and images for: The Need for Testing—The Exercise Challenge Test to Disentangle Causes of Childhood Exertional Dyspnea
Source: Front Pediatr. 2022 Jan 6;9:773794. doi: 10.3389/fped.2021.773794 (PMC8770982; doi:10.3389/fped.2021.773794)

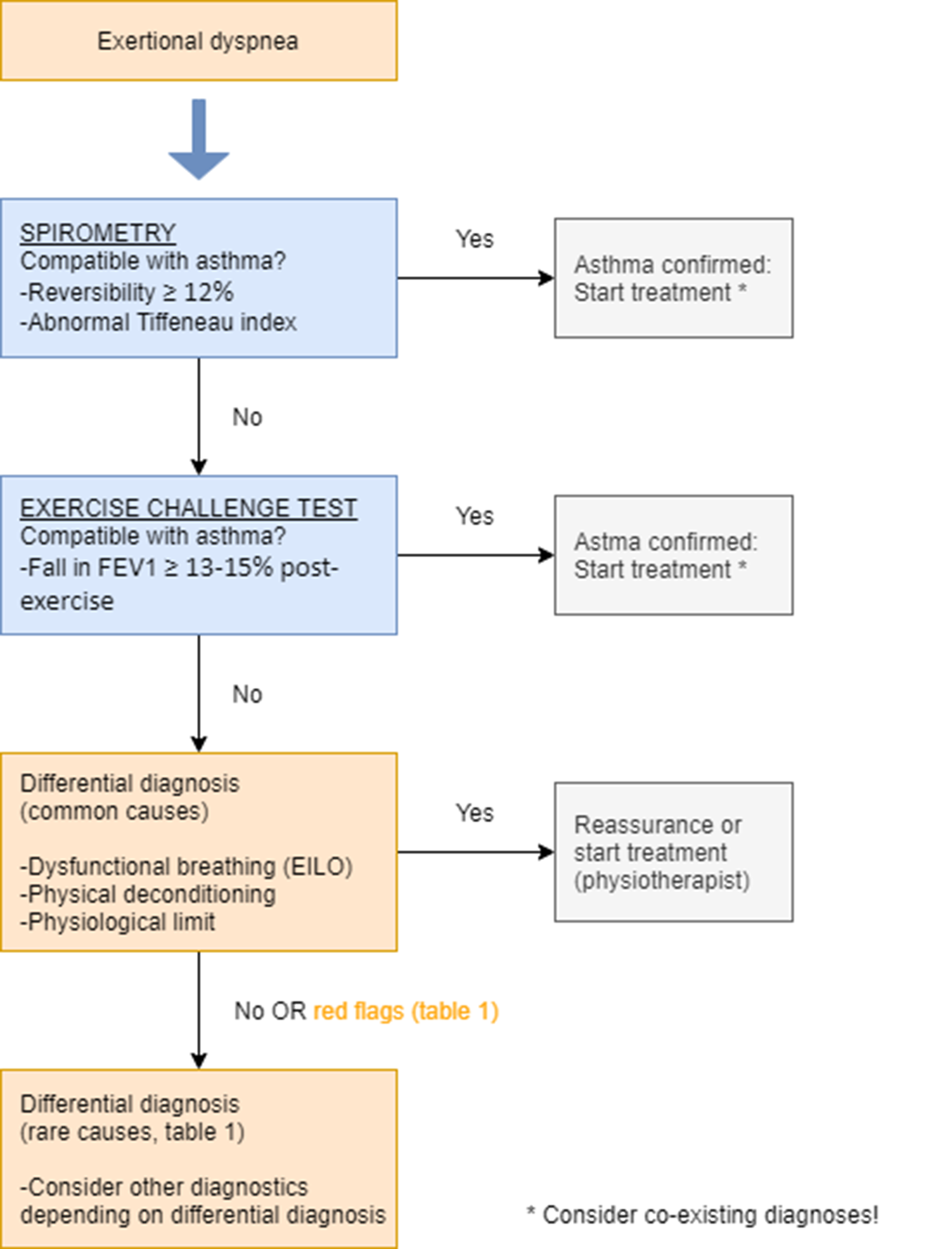

Supplement: Supplementary Figure 1 — Diagnostic pathway for childhood exertional dyspnea. [file Image_1.TIF]
